# Priorities and challenges for health leadership and workforce management globally: a rapid review

**DOI:** 10.1186/s12913-019-4080-7

**Published:** 2019-04-24

**Authors:** Carah Alyssa Figueroa, Reema Harrison, Ashfaq Chauhan, Lois Meyer

**Affiliations:** School of Public Health and Community Medicine, University of New South Wales, UNSW, Sydney, 2052 Australia

**Keywords:** Health service management, Health leadership, Workforce, Global health, Challenges, Priorities

## Abstract

**Background:**

Health systems are complex and continually changing across a variety of contexts and health service levels. The capacities needed by health managers and leaders to respond to current and emerging issues are not yet well understood. Studies to date have been country-specific and have not integrated different international and multi-level insights. This review examines the current and emerging challenges for health leadership and workforce management in diverse contexts and health systems at three structural levels, from the overarching macro (international, national) context to the meso context of organisations through to the micro context of individual healthcare managers.

**Methods:**

A rapid review of evidence was undertaken using a systematic search of a selected segment of the diverse literature related to health leadership and management. A range of text words, synonyms and subject headings were developed for the major concepts of global health, health service management and health leadership. An explorative review of three electronic databases (MEDLINE®, Pubmed and Scopus) was undertaken to identify the key publication outlets for relevant content between January 2010 to July 2018. A search strategy was then applied to the key journals identified, in addition to hand searching the journals and reference list of relevant papers identified. Inclusion criteria were independently applied to potentially relevant articles by three reviewers. Data were subject to a narrative synthesis to highlight key concepts identified.

**Results:**

Sixty-three articles were included. A set of consistent challenges and emerging trends within healthcare sectors internationally for health leadership and management were represented at the three structural levels. At the macro level these included societal, demographic, historical and cultural factors; at the meso level, human resource management challenges, changing structures and performance measures and intensified management; and at the micro level shifting roles and expectations in the workplace for health care managers.

**Conclusion:**

Contemporary challenges and emerging needs of the global health management workforce orient around efficiency-saving, change and human resource management. The role of health managers is evolving and expanding to meet these new priorities. Ensuring contemporary health leaders and managers have the capabilities to respond to the current landscape is critical.

## Background

Health systems are increasingly complex; encompassing the provision of public and private health services, primary healthcare, acute, chronic and aged care, in a variety of contexts. Health systems are continually evolving to adapt to epidemiological, demographic and societal shifts. Emerging technologies and political, economic, social, and environmental realities create a complex agenda for global health [1]. In response, there has been increased recognition of the role of non-state actors to manage population needs and drive innovation. The concept of ‘collaborative governance,’ in which non-health actors and health actors work together, has come to underpin health systems and service delivery internationally [1] in order to meet changing expectations and new priorities. Seeking the achievement of universal health coverage (UHC) and the Sustainable Development Goals (SDGs), particularly in low- and middle-income countries, have been pivotal driving forces [2]. Agendas for change have been encapsulated in reforms intended to improve the efficiency, equity of access, and the quality of public services more broadly [1, 3].

The profound shortage of human resources for health to address current and emerging population health needs across the globe was identified in the World Health Organization (WHO) landmark publication ‘Working together for health’ and continues to impede progress towards the SDGs [4]. Despite some improvements overall in health workforce aggregates globally, the human resources for health challenges confronting health systems are highly complex and varied. These include not only numerical workforce shortages but imbalances in skill mix, geographical maldistribution, difficulty in inter-professional collaboration, inefficient use of resources, and burnout [2, 5, 6]. Effective health leadership and workforce management is therefore critical to addressing the needs of human resources within health systems and strengthening capacities at regional and global levels [4, 6–8].

While there is no standard definition, health leadership is centred on the ability to identify priorities, provide strategic direction to multiple actors within the health system, and create commitment across the health sector to address those priorities for improved health services [7, 8]. Effective management is required to facilitate change and achieve results through ensuring the efficient mobilisation and utilisation of the health workforce and other resources [8]. As contemporary health systems operate through networks within which are ranging levels of responsibilities, they require cooperation and coordination through effective health leadership and workforce management to provide high quality care that is effective, efficient, accessible, patient-centred, equitable, and safe [9]. In this regard, health leadership and workforce management are interlinked and play critical roles in health services management [7, 8].

Along with health systems, the role of leaders and managers in health is evolving. Strategic management that is responsive to political, technological, societal and economic change is essential for health system strengthening [10]. Despite the pivotal role of health service management in the health sector, the priorities for health service management in the global health context are not well understood. This rapid review was conducted to identify the current challenges and priorities for health leadership and workforce management globally.

## Methods

This review utilised a rapid evidence assessment (REA) methodology structured using the Preferred Reporting Items for Systematic Reviews and Meta-Analyses (PRISMA) checklist [11]. An REA uses the same methods and principles as a systematic review but makes concessions to the breadth or depth of the process to address key issues about the topic under investigation [12–14]. An REA provides a balanced assessment of what is already known about an issue, and the strength of evidence. The narrower research focus, relative to full systematic reviews, make REAs helpful for systematically exploring the evidence around a particular issue when there is a broad evidence base to explore [14]. In the present review, the search was limited to contemporary literature (post 2010) selected from leading health service management and global health journals identified from exploring major electronic databases.

### Search strategy

#### Phase 1

An explorative review of three core databases in the area of public health and health services (MEDLINE®, Pubmed and Scopus) was undertaken to identify the key publication outlets for relevant content. These databases were selected as those that would be most relevant to the focus of the review and have the broadest range of relevant content. A range of text words, synonyms and subject headings were developed for the major constructs: global health, health service management and health leadership, priorities and challenges. Regarding health service management and health leadership, the following search terms were used: “healthcare manag*” OR “health manag*” OR “health services manag*” OR “health leader*”. Due to the large volume of diverse literature generated, a systematic search was then undertaken on the key journals that produced the largest number of relevant articles. The journals were selected as those identified as likely to contain highly relevant material based on an initial scoping of the literature.

#### Phase 2

Based on the initial database search, a systematic search for articles published in English between 1 January 2010 and 31 July 2018 was undertaken of the current issues and archives of the following journals: Asia-Pacific Journal of Health Management; BMC Health Services Research; Healthcare Management Review; International Journal of Healthcare Management; International Journal of Health Planning and Management; Journal of Healthcare Management; Journal of Health Organisation and Management; and, Journal of Health Management. Hand-searching of reference lists of identified papers were also used to ensure that major relevant material was captured.

### Study selection and data extraction

**Fig. 1:**
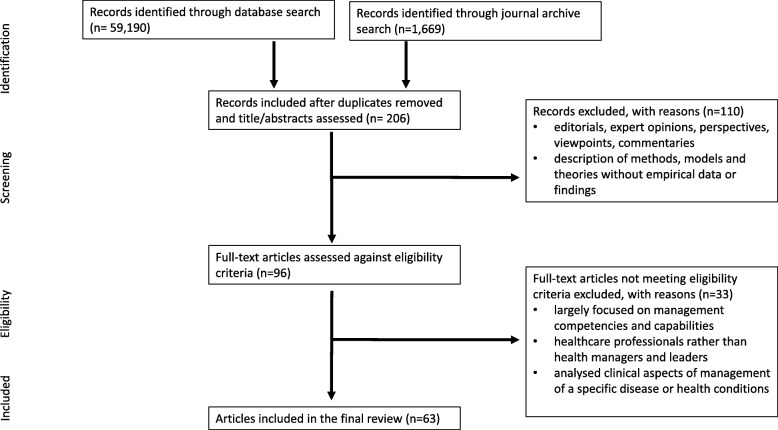
PRISMA flow chart of the literature search, identification, and inclusion for the review

Results were merged using reference-management software (Endnote) and any duplicates removed. The first author (CF) screened the titles and abstracts of articles meeting the eligibility criteria (Table 1). Full-text publications were requested for those identified as potentially relevant. The inclusion and exclusion criteria were then independently applied by two authors. Disagreements were resolved by consensus or consultation with a third person, and the following data were extracted from each publication: author(s), publication year, location, primary focus and main findings in relation to the research objective. Sixty-three articles were included in the final review. The selection process followed the PRISMA checklist [11] as shown in Fig. 1.

**Table 1:** Eligibility criteria for selecting studies for the review

Inclusion criteria	Exclusion criteria
Date and language limits	
Between 1 January 2010 and 31 July 2018, English	Pre-2010, Non-English
Type of publication	
Peer-reviewed research articles	Editorials, expert opinions, perspectives, viewpoints, commentaries and other articles where an abstract and methods are not described
Study design	
All study designs	Description of methods, models and theories without empirical data or findings
Content	
Reported outcomes relating to global challenges, issues, needs, with reference to health management or leadership.	Analysis of the clinical aspects of management of a specific disease or health condition. Specifically focused on competencies and capabilities in managers and leaders
Target group	
Managers and leaders in health	Other health care professionals

### Data extraction and analysis

A narrative synthesis was used to explore the literature against the review objective. A narrative synthesis refers to “an approach to the systematic review and synthesis of findings from multiple studies that relies primarily on the use of words and text to summarise and explain the findings of the synthesis” [15]. Firstly, an initial description of the key findings of included studies was drafted. Findings were then organised, mapped and synthesised to explore patterns in the data.

## Results

### Search results

A total of 63 articles were included; Table 2 summarizes the data extraction results by region and country. Nineteen were undertaken in Europe, 16 in North America, and one in Australia, with relatively fewer studies from Asia, the Middle East, and small island developing countries. Eighteen qualitative studies that used interviews and/or focus group studies [16–32] were identified. Other studies were quantitative [33–39] including the use of questionnaires or survey data, or used a mixed-method approach [40–44]. Other articles combined different types of primary and secondary data (key informant interviews, observations, focus groups, questionnaire/survey data, and government reports). The included literature also comprised 28 review articles of various types that used mixed data and bibliographic evidence.

**Table 2:** Literature included in this review, by context and their references

Context	Literature describing challenges and emerging trends
International^a^	[40, 56, 61, 68, 69, 73]
Europe	[16, 62, 63, 74]
Africa	[46]
Australia	[59, 67]
Botswana	[80]
Brazil	[17, 41]
Canada	[18–20, 81]
Caribbean	[47]
Costa Rica	[72]
Finland	[21]
Germany	[33]
India	[22, 34, 48]
Indonesia	[35]
Iran	[23, 24, 49]
Ireland	[53]
Italy	[36]
Netherlands	[55]
Norway	[71]
Pacific Islands	[50]
Portugal	[25]
Spain	[65]
South Korea	[57]
South Africa	[51]
Sweden	[26, 70]
Switzerland	[37]
Syria	[52]
Tanzania	[27]
Thailand	[28]
Turkey	[64]
United Kingdom (UK)	[29, 42, 43, 75]
United States of America (USA)	[30–32, 38, 39, 44, 54, 58, 60, 66, 82, 83]

^a^refers to more than one country situated in different regions including Europe, North America, Asia, and Africa

### Key challenges and emerging trends

A set of challenges and emerging trends were identified across healthcare sectors internationally. These were grouped at three levels: 1) macro, system context (society, demography, technology, political economy, legal framework, history, culture), 2) meso, organisational context (infrastructure, resources, governance, clinical processes, management processes, suppliers, patients), and 3) micro context related to the individual healthcare manager (Table 3). This multi-levelled approach has been used in previous research to demonstrate the interplay between different factors across different levels, and their direct and indirect reciprocal influences on healthcare management policies and practices [45].

**Table 3:** Overview and types of challenges and emerging trends for healthcare managers internationally within the included literature

Level	Challenge or emerging trend	Aspects of the challenge or emerging trend	References
Societal and system-wide (macro)	Demographic and epidemiological transitions	Population growth	[34, 47, 48, 61]
		Ageing populations	[21, 47, 49, 53, 57, 61, 69]
		Rise in chronic, non-communicable disease and lifestyle-related health issues	[21, 46–52, 56]
		High disease burdens and poor health indicators	[46, 47, 51]
	Growing and shifting supply and demand patterns	More patients with complex needs requiring multiple healthcare providers	[21, 46, 54, 55, 83]
		Hospital capacity issues	[50, 53]
		More knowledgeable and health-literate consumers	[34, 53, 54]
		Higher expectations from healthcare organizations (value-for-money)	[16, 34, 43, 53, 57, 60]
		Increasing dissatisfaction with healthcare system	[61]
		Greater treatment affordability, increased medical tourism, growing health insurance use, rising incomes	[48]
		Inequalities in access to healthcare	[51, 72]
	Advances in science and technology	New Information and communication Technology (ICT) systems	[47, 48, 53, 54, 57, 69, 83]
		Innovations in healthcare services and delivery (electronic medical records, telemedicine, internet-based care, hospital and ward redesign)	[47, 54, 56–58]
		New categories or specialization of service providers	[54, 83]
		Greater integration and interdisciplinary teams and collaborative healthcare practice	[54, 55]
	Political and economic change	Adapting to changes in government and health sector reforms	[18, 19, 24, 28, 31, 47, 53, 54, 59, 60]
		Decentralisation of healthcare	[24, 27, 35, 59, 72]
		Budget constraints, measures to avoid deficits	[16, 19, 53, 60, 61]
		Disconnection between population needs and resource allocation	[23, 27, 40, 47, 57, 72]
		Lack of or increasing collaboration between governments, health providers, community representatives and other stakeholders to address the needs of healthcare systems	[27, 40, 49]
		Shifting to patient-focused care; greater attention to community health and addressing social determinants of health	[16, 21, 28, 32, 34, 38, 53–55, 58]
	Corporatisation and privatisation	Emergence of new business models for healthcare; Public–Private Partnership (PPP) models	[22, 48, 54, 59, 62, 63]
		Move from independent health organisations to large, networked health systems	[22, 59, 62]
		High or uneven demand for specialist tertiary care	[22, 49]
		Growth of the private sector; competition for health professionals	[22, 34, 35, 57, 61, 62]
	Increasing costs	Healthcare costs	[21, 22, 53, 61, 64, 69]
		Managerial costs	[34, 64]
		Costs associated with developing new programmes	[19, 47, 53]
	Crises in human resources for health	Shortage of trained health personnel, out-migration of skilled health workers	[23, 25, 41, 46, 47, 50, 51, 61]
		Lack of effective retention strategies and poor working conditions	[46, 80]
		Challenge to maintain health services with appropriate skill mixes	[35, 46, 47, 51, 61]
		Limited resources and health infrastructure and their maintenance	[46, 47, 50, 72]
		Deficiencies in health information systems	[23, 25, 49]
Organisational (meso)	Human resource management challenges	Inefficiency and insufficiencies in provision of health services and use of resources; increased demands for efficiency and cost-cutting	[18, 21, 49, 53, 57, 61, 63–65]
		Barriers to implementing lean healthcare: outsourcing hospital activities, limited knowledge of lean	[17, 21]
		Inadequate planning and performance evaluation systems; poor talent identification; poor deployment and underutilization of staff	[23, 25, 28, 30, 43, 49, 69, 72, 80]
		Lack of support and opportunities in management training and leadership development within organisations	[22, 26, 28, 31, 41, 42, 46, 47, 67, 82]
		Poor quality of services or concerns of declining quality; poor culture regarding patient safety	[18, 33, 35, 46, 61, 69]
	Changes in organisational structures and measures	Dominant hierarchical culture	[21, 22, 36, 43, 54, 63, 64, 72]
		Selective recruitment into leadership positions; need for robust succession planning and management	[44, 66, 67]
		Excessive bureaucracy or lack of transparency in organisational rules and processes	[21, 24, 30, 64, 67]
		Inadequate systems to prevent and control healthcare associated infections (HAIs)	[53, 68]
		Target-driven approach to performance measurement	[61]
		Fee-for-service payment models encouraging volume not quality of care	[18, 23, 57, 61]
		Value-based payment models, other new payment models	[24, 49, 62, 69, 70, 72, 83]
	Intensification of front-line and middle management work	Broad responsibility; balancing clinical, teaching, research and management roles	[22, 28, 29, 42, 53, 64, 70, 81]
		Long working hours, unpredictable work patterns, tight deadlines, stress and reduced productivity	[22, 29, 37, 42, 51]
		Difficulties of middle-level and front-line managers to operationalise executive strategic directions and initiatives (lack of incentives, lack of support, resource constraints, conflict between organisational priorities and employees’ own goals and values)	[16, 21, 24, 26, 30, 31, 37, 42, 53, 65, 72, 81]
		Informal and shared leadership in the front-line in the absence of formal management	[20]
Individual (micro)	Shifting health manager role	No universal standard definition for a health manager nor defined competency standards	[28, 59]
		Lack of transparency and accountability	[24, 28, 30, 31, 67]
		Increasing dual clinician and manager and leadership roles	[18, 28, 53, 63, 70, 71, 74, 75]
		More physicians becoming senior healthcare managers	[39, 63, 64]
		More non-physician health managers, new types of professional healthcare managers	[73, 74]

#### Societal and system-wide (macro)

Population growth, ageing populations, and increased disease burdens are some of the common trends health systems are facing globally. Developing and developed countries are going through demographic and epidemiological transitions; people are living longer with increasing prevalence of chronic diseases requiring health managers and leaders to adjust to shifting healthcare needs at the population level, delivering preventative and long-term care beyond acute care. Countries in Africa, Europe, the Pacific Islands, Middle East, Asia and Caribbean are seeing an increase in number of patients with non-communicable diseases and communicable diseases [21, 46–52].

Although many countries have similar emerging health system concerns, there are some differences in the complexities each country faces. For many small countries, outmigration, capacity building and funding from international aid agencies are affecting how their health systems operate, while in many larger countries, funding cuts, rise in private health insurance, innovations, and health system restructuring are major influences [21, 34, 50, 53, 54]. In addition, patients are increasingly health literate and, as consumers, expect high-quality healthcare [34, 53, 54]. However, hospitals and healthcare systems are lacking capacity to meet the increased demand [16, 34, 43].

Scientific advances have meant more patients are receiving care across the health system. It is imperative to have processes for communication and collaboration between different health professionals for high-quality care. However, health systems are fragmented; increasing specialisation is leading to further fragmentation and disassociation [31, 54, 55]. Adoption of technological innovations also require change management, hospital restructure, and capacity building [56–58].

Changes in health policies and regulations compound the challenge faced by healthcare managers and leaders to deliver high quality care [53, 54, 59]. Political reforms often lead to health system restructuring requiring change in the values, structures, processes and systems that can constrain how health managers and leaders align their organisations to new agendas [24, 28, 31, 60]. For example, the distribution of health services management to local authorities through decentralisation has a variable impact on the efficacy and efficiency of healthcare delivery [24, 27, 35, 59].

Governments’ decisions are often made focusing on cost savings, resulting in budgetary constraints within which health systems must operate [16, 19, 53, 61]. Although some health systems have delivered positive results under such constraint [53], often financial resource constraints can lead to poor human and technical resource allocation, creating a disconnect between demand and supply [23, 27, 40, 47, 57]. To reduce spending in acute care, there is also a push to deliver health services in the community and focus on social determinants of health, though this brings further complexities related to managing multiple stakeholder collaborations [27, 32, 34, 38, 40, 49, 55].

Due to an increase in demand and cost constraints, new business models are emerging, and some health systems are resorting to privatisation and corporatisation [22, 48, 62]. This has created competition in the market, increased uptake of private health insurance and increased movement of consumers between various organisations [22, 48]. Health managers and leaders need to keep abreast with continuously changing business models of care delivery and assess their impact [59, 62]. The evolving international health workforce, insufficient numbers of trained health personnel, and maintaining and improving appropriate skill mixes comprise other important challenges for managers in meeting population health needs and demands (Table 3).

#### Organisational level (meso)

At the organisation level, human resource management issues were a central concern. This can be understood in part within the wider global human resources for health crisis which has placed healthcare organisations under intense pressure to perform. The evidence suggests healthcare organisations are evolving to strengthen coordination between primary and secondary care; there is greater attention to population-based perspectives in disease prevention, interdisciplinary collaboration, and clinical governance. These trends are challenged by the persistence of bureaucratic and hierarchical cultures, emphasis on targets over care quality, and the intensification of front-line and middle-management work that is limiting capacity.

Healthcare managers and leaders also face operational inefficiencies in providing primary health and referral services to address highly complex and shifting needs which often result in the waste of resources [49, 63, 64]. Considering the pace of change, organisations are required to be flexible and deliver higher quality care at lower cost [21, 53, 65]. To achieve this, many organisations in developing and developed countries alike are adopting a lean model [17, 21]. However, there are challenges associated with ensuring sustainability of the lean system, adjusting organisational hierarchies, and improving knowledge of the lean model, especially in developing countries [17, 21].

Healthcare organisations require various actors with different capabilities to deliver high quality care. However, a dominant hierarchical culture and lack of collaborative and distributed culture can limit the performance of healthcare organisations [22, 36, 54]. In addition, considering high turnover of executive leadership, healthcare organisations often rely on external talent for succession management which can reduce hospital efficiency [44, 66]. Other contributors to weakened hospital performance include: the lack of allocative efficiency and transparency [24, 30, 64, 67]; poor hospital processes that hamper the development of effective systems for the prevention and control of hospital acquired infections (HAIs) [53, 68]; and, payment reforms such as value-based funding and fee-for-service that encourage volume [18, 23, 24, 61, 62, 69, 70].

Managerial work distribution within organisations is often not clearly defined, leading to extra or extreme work conditions for middle and front-line managers [29, 42, 53, 70]. Unregulated and undefined expectations at the organisation level leads to negative effects such as stress, reduced productivity, and unpredictable work hours, and long-term effects on organisational efficiency and delivery of high quality care [22, 28, 29, 37, 42, 51, 71]. Furthermore, often times front-line clinicians are also required to take the leadership role in the absence of managers without proper training [20]. Despite this, included studies indicate that the involvement of middle and front-line managers in strategic decision-making can be limited due to various reasons including lack of support from the organisation itself and misalignment of individual and organisational goals [16, 26, 31, 72].

#### Individual level (micro)

Worldwide, middle and front-line health managers and leaders are disproportionately affected by challenges at the system and organisational level, which has contributed to increasing and often conflicting responsibilities. Some countries are experiencing a growth in senior health managers with a clinical background, while in other countries, the converse is apparent. Indistinct organisational boundaries, increasing scope of practice, and lack of systemic support at policy level are leaving healthcare managers with undefined roles [28, 59]. Poorly defined roles contribute to reduced accountability, transparency, autonomy, and understanding of responsibilities [24, 30, 31, 67]. Studies also indicate a lack of recognition of clinical leaders in health organisations and inadequate training opportunities for them as such [20, 67].

The number of hybrid managers (performing clinical and managerial work concurrently) in developed countries is increasing, with the perception that such managers improve the clinical governance of an organization. In contrast, the number of non-clinical managers in many developing countries appears to be increasing [63, 73–75]. Included studies suggest this approach does not necessarily improve manager-clinical professional relationships or the willingness of clinicians becoming managers, limiting their participation in strategic decisions [28, 70, 71, 74].

## Discussion

This rapid review highlights the current global climate in health service management, the key priority areas, and current health management approaches being utilised to address these. The multitude of issues emerging demonstrate the complex and evolving role of health service management in the wider complex functioning of health systems globally in a changing healthcare landscape. Key themes of achieving high quality care and sustainable service delivery were apparent, often evidenced through health reforms [5]. The influence of technological innovation in both its opportunities and complexities is evident worldwide. In the context of changing healthcare goals and delivery approaches, health management is seeking to professionalise as a strategy to build strength and capacity. In doing so, health managers are questioning role scope and the skills and knowledge they need to meet the requirements of the role.

### Global challenges facing health management

Understanding how the features of the macro, meso and micro systems can create challenges for managers is critical [19]. With continual healthcare reform and increasing health expenditure as a proportion of GDP, distinct challenges are facing high-income Organisation for Economic Co-operation and Development (OECD) countries, middle-income rapidly-developing economies, and low-income, resource-limited countries. Reforms, especially in OECD countries, have been aimed at controlling costs, consolidating hospitals for greater efficiencies, and reconfiguring primary healthcare [1, 76]. The changing business models for the delivery of care have wider implications for the way in which health managers conceptualise healthcare delivery and the key stakeholders [59], for example, the emerging role of private healthcare providers and non-health actors in public health. Changes to the business model of healthcare delivery also has implications for the distribution of power amongst key actors within the system. This is evident in the evolved role of general practitioners (GPs) in the UK National Health Service as leaders of Clinical Commissioning Groups (CCGs). Commissioning requires a different skill set to clinical work, in terms of assessing financial data, the nature of statutory responsibilities, and the need to engage with a wider stakeholder group across a region to plan services [77]. With new responsibilities, GPs have been required to quickly equip themselves with new management capabilities, reflecting the range of studies included in this review around clinician managers and the associated challenges [18, 28, 53, 63, 70, 71, 74, 75].

Central to the role of healthcare managers is the ability to transition between existing and new cultures and practices within healthcare delivery [59]. Bridging this space is particularly important in the context of increasingly personalized and technologically-driven healthcare delivery [54]. While advances in knowledge and medical technologies have increased capability to tackle complex health needs, the integration of innovations into existing healthcare management practices requires strong change management [73]. Health leaders and managers need to be able to rapidly and continually assess the changes required or upon them, the implications, and to transform their analysis into a workable plan to realise change [10]. Focusing only on the clinical training of health professionals rather than incorporating managerial and leadership roles, and specifically, change management capability may limit the speed and success of innovation uptake [22].

### Implications

Our findings highlight the implications of current priorities within the health sector for health management practice internationally; key issues are efficiency savings, change management and human resource management. In the context of efficiency approaches, health system and service managers are facing instances of poor human and technical resource allocation, creating a disconnect between demand and supply. At the service delivery level, this has intensified and varied the role of middle managers mediating at two main levels. The first level of middle-management is positioned between the front-line and C-suite management of an organisation. The second level of middle-management being the C-suite managers who translate regional and/or national funding decisions and policies into their organisations. Faced with increasing pace of change, and economic and resource constraints, middle managers across both levels are now more than ever exposed to high levels of stress, low morale, and unsustainable working patterns [29]. Emphasis on cost-saving has brought with it increased attention to the health services that can be delivered in the community and the social determinants of health. Connecting disparate services in order to meet efficiency goals is a now a core feature of the work of many health managers mediating this process.

Our findings also have implications for the conceptualisation of healthcare management as a profession. The scale and increasing breadth of the role of health leaders and managers is evident in the review. Clarifying the professional identity of ‘health manager’ may therefore be a critical part of building and maintaining a robust health management workforce that can fulfil these diverse roles [59]. Increasing migration of the healthcare workforce and of population, products and services between countries also brings new challenges for healthcare. In response, the notion of transnational competence among healthcare professionals has been identified [78]. Transnational competence progresses cultural competence by considering the interpersonal skills required for engaging with those from diverse cultural and social backgrounds. Thus, transnational competence may be important for health managers working across national borders. A key aspect of professionalisation is the education and training of health managers. Our findings provide a unique and useful theoretical contribution that is globally-focused and multi-level to stimulate new thinking in health management educators, and for current health leaders and managers. These findings have considerable practical utility for managers and practitioners designing graduate health management programs.

### Limitations

Most of the studies in the field have focused on the Anglo-American context and health systems. Notwithstanding the importance of lessons drawn from these health systems, further research is needed in other regions, and in low- and middle-income countries in particular [79]. We acknowledge the nuanced interplay between evidence, culture, organisational factors, stakeholder interests, and population health outcomes. Terminologies and definitions to express global health, management and leadership vary across countries and cultures, creating potential for bias in the interpretation of findings. We also recognise that there is fluidity in the categorisations, and challenges arising may span multiple domains. This review considers challenges facing all types of healthcare managers and thus lacks discrete analysis of senior, middle and front-line managers. That said, managers at different levels can learn from one another. Senior managers and executives may gain an appreciation for the operational challenges that middle and front-line managers may face. Middle and front-line managers may have a heightened awareness of the more strategic decision-making of senior health managers. Whilst the findings indicate consistent challenges and needs for health managers across a range of international contexts, the study does not capture country-specific issues which may have consequences at the local level. Whilst a systematic approach was taken to the literature in undertaking this review, relevant material may have been omitted due to the limits placed on the rapid review of the vast and diverse health management literature. The inclusion of only materials in English language may have led to further omissions of relevant work.

## Conclusion

Health managers within both international and national settings face complex challenges given the shortage of human resources for health worldwide and the rapid evolution of national and transnational healthcare systems. This review addresses the lack of studies taking a global perspective of the challenges and emerging needs at macro (international, national and societal), meso (organisational), and micro (individual health manager) levels. Contemporary challenges of the global health management workforce orient around demographic and epidemiological change, efficiency-saving, human resource management, changing structures, intensified management, and shifting roles and expectations. In recognising these challenges, researchers, management educators, and policy makers can establish global health service management priorities and enhance health leadership and capacities to meet these. Health managers and leaders with adaptable and relevant capabilities are critical to high quality systems of healthcare delivery.

## Abbreviations

CCGs: Clinical Commissioning Groups
GPs: General practitioners
HAIs: Hospital acquired infections
OECD: Organisation for Economic Co-operation and Development
PRISMA: Preferred Reporting Items for Systematic Reviews and Meta-Analyses
REA: Rapid evidence assessment
SDGs: Sustainable Development Goals
UHC: Universal health coverage
WHO: World Health Organization
